# Preponderance of *bla*_KPC_-Carrying Carbapenem-Resistant Enterobacterales Among Fecal Isolates From Community Food Handlers in Kuwait

**DOI:** 10.3389/fmicb.2021.737828

**Published:** 2021-10-14

**Authors:** Ola H. Moghnia, Vincent O. Rotimi, Noura A. Al-Sweih

**Affiliations:** Department of Microbiology, Faculty of Medicine, Kuwait University, Kuwait City, Kuwait

**Keywords:** *bla*_KPC_, carbapenem-resistant Enterobacterales, food handlers, rectal colonization, molecular characterization

## Abstract

Carbapenem-resistant Enterobacterales (CRE) are pathogens that have been found in several countries, with a significant public health concern. Characterizing the mode of resistance and determining the prevailing clones are vital to the epidemiology of CRE in our community. This study was conducted to characterize the molecular mode of resistance and to determine the clonality of the CRE fecal isolates among community food handlers (FHs) vs. infected control patients (ICPs) in Kuwait. Fecal CRE isolates obtained from FHs and ICPs from September 2016 to September 2018 were analyzed for their resistance genes. Gene characterization was carried out by polymerase chain reaction (PCR) assays and sequencing. Clonality of isolates was established by multilocus sequence typing (MLST). Of the 681 and 95 isolates of the family Enterobacterales isolated from FHs and ICPs, 425 (62.4%) and 16 (16.8%) were *Escherichia coli*, and 18 (2.6%) and 69 (72.6%) were *Klebsiella pneumoniae*, respectively. A total of 36 isolates were CRE with a prevalence of 5.3% among FH isolates and 87 (91.6%) among the ICPs. Of these, carbapenemase genes were detected in 22 (61.1%) and 65 (74.7%) isolates, respectively (*p* < 0.05). The detected specific genes among FHs and ICPs were positive for *bla*_KPC_ 19 (86.4%) and 35 (40.2%), and *bla*_OXA_ 10 (45.5%) and 59 (67.8%), in addition to *bla*_NDM_ 2 (9.1%) and 32 (36.8%), respectively. MLST assays of the *E. coli* and *K. pneumoniae* isolates revealed considerable genetic diversity and polyclonality as well as demonstrated multiple known ST types and eight novel sequence types. The study revealed a relatively high number of CRE harboring predominantly *bla*_KPC_-mediated CRE among the community FH isolates vs. predominant *bla*_OXA_ genes among the ICPs. Those heterogeneous CRE isolates raise concerns and mandate more efforts toward molecular surveillance. A multinational study is recommended to monitor the spread of genes mediating CRE in the community of Arabian Peninsula countries.

## Introduction

Carbapenems have been used as effective and drugs of choice over the years to treat life-threatening nosocomial infections, particularly bloodstream infections, transplant-related infections, ventilator-associated infections, and infections in hospitalized patients in intensive care units, in addition to infections caused by extended-spectrum β-lactamases and AmpC-producing species of the family Enterobacterales. With an increase in the number of people exposed to antibiotics, the intestinal microflora remains a selective pressure for multidrug-resistant (MDR) bacteria due to the milieu of antibiotics consumed by patients. Enterobacterales are inhabitants of the intestinal flora and are among the most common human pathogens causing community and healthcare-associated infections. They have the propensity to acquire genetic material through horizontal gene transfer. The emergence of carbapenem-resistant Enterobacterales (CRE) is an increasing threat to global health. The primary mechanism of resistance is the production of carbapenemases. In the past years, the worldwide spread of CRE and the mechanism of resistance in these isolates attracted much attention because of their rapid global transmission and limited therapeutic options for the infections caused, posing an urgent threat to the efficacy of carbapenem antibiotics. KPC genes have spread internationally among Gram-negative bacteria in China, Greece, Italy, Poland, Colombia, Argentina, Brazil, and some states in the United States but not in Kuwait (Munoz-Price et al., 2013; Stoesser et al., 2017; Cienfuegos-Gallet et al., 2019). Other carbapenemases, such as OXA-48, are present in Turkey and North Africa (Temkin et al., 2014). The Indian subcontinent is endemic for NDM variants and acts as a reservoir of these carbapenemases and other inactivating enzymes such as KPC and OXA-181 (Nordmann and Poirel, 2014). A few studies have been conducted in our hospitals to determine the prevalence and burden of CRE in the country. A report documenting cases of nosocomial acquisition of two NDM-1 producing *Klebsiella pneumoniae* isolates in Indian and indigenous Kuwaiti patients, who had no history of travel, was published in 2012 by Jamal et al. (2012). Other reports have highlighted the emergence of VIM-4 and NDM-1-producing Enterobacterales in Kuwait (Jamal et al., 2013, 2015). Food handlers (FHs) who are considered an important link in the chain from farm to fork are at high risk of being CRE colonized or infected. Those unrecognized workers may serve as a reservoir for CRE transmission and play an essential role in spreading these organisms in community as well as healthcare settings. At the same time, FHs have an important integral part in the community in preventing food contamination. This largely depends on their health status and hygiene practices, which may occur at any point in the journey from the producer to the consumer. Studies have found that poor personal hygiene could be a potential source of infections and may serve as a reservoir of genes for antimicrobial resistance in organisms (Luo et al., 2011). Early detection of carriers or colonizers facilitates the rapid establishment of contact precautions to prevent acquiring CRE. The emergence of CRE as a global problem was extensively studied in healthcare settings. Delineation of genes encoding carbapenemase production in CRE colonizing the rectum of FHs has never been explored in community settings. Therefore, the present study aimed to evaluate the prevalence of genes mediating carbapenemase production in CRE isolates circulating among FH population in the community of Kuwait.

## Materials and Methods

### Study Design

This study was conducted between September 2016 and September 2018 among FHs working catering establishments in the community. In addition, clinically proven infected control patients (ICPs) were admitted to four teaching hospitals, including Mubarak Al-Kabeer (MAK), Farwaniya (FAH), Ibn-Sina (ISH), and Al Babtain (BabH) Hospitals, and were investigated as the control group. A descriptive analysis of demographic characteristics and predisposing factors of a healthy population of volunteer FHs was performed previously (Moghnia et al., 2021a).

### Bacterial Isolates

Non-duplicate 405 fecal samples and 92 rectal swabs were collected from FHs and ICPs, respectively. Fecal samples were prospectively self-collected by FHs in privacy, following instructions, in a clean, dry screw-top container. Then, a sterile cotton-wool swab with 5 ml of Amies gel transport medium (Copan, Brescia, Italy) was dipped into the stool specimen collected from each of the FHs. In addition, rectal swabs were collected from ICPs. Then, swabs were immediately inoculated on freshly prepared MacConkey agar and blood agar plates (Oxoid, Basingstoke, Hants, United Kingdom). The plates were incubated in an aerobic incubator (Gallenkamp, Widnes, England) at 37°C for 18–24 h. A pure colony of CRE isolate was selected from each sample and cultured into a new MacConkey agar plate to obtain pure growth, and then the plate was incubated at 37°C for 18–24 h.

CRE isolates were identified to the species level by the Gram-negative identification card on VITEK 2 ID automated System (bioMérieux, Marcy l’Etoile, France). The minimum inhibitory concentrations (MICs) of the antibiotics tested that inhibited 90% (MIC90) and 50% (MIC50) of the isolates were determined using both E-test (bioMérieux, Marcy l’Etoile, France) and agar dilution methods according to the manufacturer’s instruction as previously described (Moghnia et al., 2021b) according to the Clinical Laboratory Standards Institute interpretative criteria. Carbapenem resistance isolate was defined as an Enterobacterales isolate that was non-susceptible to at least one of the carbapenems with MIC of > 0.5 μg/ml for ertapenem, or > 1 μg/ml for imipenem and meropenem (CLSI [Clinical and Laboratory Standard Institute], 2018).

### Indirect Carbapenemase Test

The carbapenemase production (OXA, KPC, NDM, IMP, and VIM) of CRE isolates from FHs and infected control group was investigated with indirect carbapenemase test MAST^®^ICT (Mast Diagnostic, France) according to the manufacturer’s instruction.

### Polymerase Chain Reaction Analysis of Carbapenemase and Sequencing

The presence of genes encoding the carbapenemases was detected by PCR amplification assays using previously published primers (Sigma-Aldrich, Darmstadt, Germany) designed to detect *bla*_KPC_, *bla*_OXA_ (Poirel et al., 2011), *bla*_NDM_ (Ellem et al., 2011), *bla*_VIM_, *bla*_IMP_ (Toleman et al., 2003), and *bla*_SIM_ (Lee et al., 2003). These primers as well as the PCR cycling conditions are reported in Table 1. Sequencing of the amplicons was performed to identify *bla* variants using the GeneAmp PCR system 9700 by cycle sequencing with ABI Prism BigDye terminator V3.1 Ready Reaction Cycle Sequencing Kit (Applied Biosystems, Foster City, CA, United States). The sequencing results were determined using software from the National Center for Biotechnology Information.^1^

**TABLE 1:** Primer sets used for PCR amplification of carbapenem-resistance genes and their expected amplicon size.

**Gene**	**Primer sequences**	**Amplicon size (bp)**	**PCR cycling conditions (reference)**
***bla*_KPC_**	F: CGTCTAGTTCTGCTGTCTTG R:CTTGTCATCCTTGTTAGGCG	798	Heat activation of polymerase at 95°C for 15 min, then initial denaturation at 94°C for 10 min, followed by 35 cycles of denaturation at 94°C for 30 s, annealing at 52°C for 40 s, and elongation at 72°C for 50 s, followed by a final elongation step at 72°C for 5 min (Poirel et al., 2011)
***bla*_OXA_**	F: GCGTGGTTAAGGATGAACAC R: CATCAAGTTCAACCCAACCG	438	
***bla*_NDM_**	F: CTTCCAACGGTTTGATCGTC R: ATTGGCATAAGTCGCAAT CC	206	Heat activation of polymerase at 95°C for 15 min, then initial denaturation at 95°C for 5 min, followed by 30 cycles of denaturation at 95°C for 2 min, annealing at 60°C for 1 min, and elongation at 72°C for 1 min, followed by a final elongation step at 72°C for 5 min (Ellem et al., 2011)
***bla*_IMP_**	F: ATGAGCAAGTTATCTTAGTATTC R: GCTGCAACGACTTGTTAG	765	Heat activation of polymerase at 95°C for 15 min, then initial denaturation at 95°C for 5 min, followed by 30 cycles of denaturation at 95°C for 2 min, annealing at 50°C for 1 min, and elongation at 68°C for 1 min, followed by a final elongation step at 68°C for 5 min (Toleman et al., 2003)
***bla*_VIM_**	F: TTATGGAGCAGCAACGATGT R: CGAATG CGCAGCACCAGG	621	Heat activation of polymerase at 95°C for 15 min, then initial denaturation at 95°C for 5 min, followed by 30 cycles of denaturation at 95°C for 1 min, annealing at 59°C for 1 min, and elongation at 68°C for 1 min, followed by a final elongation step at 68°C for 5 min (Toleman et al., 2003)
***bla*_SIM_**	F: TACAAGGGATTC GGCATC G R: TAATGGCCTGTTCCCATGTG	571	Heat activation of polymerase at 95°C for 15 min, then initial denaturation at 94°C for 5 min, followed by 25 cycles of denaturation at 94°C for 30 s, annealing at 52°C for 1 min, and elongation at 68°C for 1 min, followed by a final elongation step at 68°C for 5 min (Lee et al., 2003)

*The direction of the primer is indicated at the end of the primer name, as follows: F, forward (5′–3′) and R, reverse (3′–3′).KPC, Klebsiella pneumoniae carbapenemase; OXA, oxacillinase; NDM, New Delhi metallo-β-lactamase; IMP, imipenem-resistant Pseudomonas; VIM, Verona integron-encoded metallo-β-lactamase; SIM, Seoul imipenemase.*

### Multilocus Sequence Typing

Random selection of carbapenem-resistant *Escherichia coli* and *K. pneumoniae* isolates harboring carbapenemases encoding genes from FHs and ICPs were performed to understand the clonal relatedness of these isolates. Carbapenem-resistant *E. coli* (*n* = 13) isolates including FHs (*n* = 7) and ICPs (*n* = 6) isolates as well as *K. pneumoniae* isolates (*n* = 14) including FHs (*n* = 7) and ICPs (*n* = 7) isolates were assigned to multilocus sequence typing (MLST) method as described previously (Diancourt et al., 2005; Wirth et al., 2006). Allelic profiles (obtained from the pattern of allele numbers) for seven gene fragments of each isolate were obtained by comparing with corresponding allele available in MLST *E. coli* database,^2^ as well as in MLST *K. pneumoniae* database^3^ following website instruction. The sequence type (ST) of each isolate was determined by combining seven allelic profiles. The MLST data, based on the allele number for the seven gene fragments for each isolate, were used for constructing strain relatedness dendrogram by minimum spanning trees using BioNumerics software (version 6.1; Applied Maths, Kortrijk, Belgium).

Comparative goeBURST analysis was performed to determine the diversity of the *E. coli* and *K. pneumoniae* isolates against the entire *E. coli* and *K. pneumoniae* database and to reveal their relationships with all publicly available STs. STs were clustered into clonal complexes (CCs) using the goeBURST algorithm of the Phyloviz software.^4^ The goeBURST assigned each ST that shared at least five of seven identical alleles into a single CC.

### Statistical Criteria

Data were tabulated and analyzed using IBM SPSS Statistics v.25.0 (IBM Corp., Armonk, NY, United States). Significance was determined by Pearson’s chi-squared test (χ^2^) to test associations between two categorical variable CRE isolates expressing carbapenemase genes from FHs and ICPs and evaluate how likely it is that there is any observed genetic difference in the species level. The threshold for statistical significance was a *p*-value of < 0.05.

## Results

### Demographic Characteristics

A total of 405 samples and 92 samples were collected from FHs and ICPs, respectively. A total of 31 FHs and 84 ICPs were found to be colonized with CRE. The CRE colonization rates were 31/405 (7.6%) and 84/92 (91.3%) among FHs and ICPs, respectively. The non-Arab ethnic group constituted 26/31 (83.8%) of the FH CRE colonizers. The Southeast Asians represented the highest proportion among FHs, and the nationalities were as follows: 14 (45.2%), nine (29%), one (3.2%), two (6.5%), and five (16.1%) were Indians, Filipinos, Siri Lankans, Bangladeshis, and Egyptians. On the other hand, the Arab ethnic group constituted 63/84 (75%) of the ICP CRE colonizers. The top five nationalities were Kuwaitis, 36 (43%); Egyptians, nine (11%); Indians, six (7%); Jordanians, five (6%); Iranians, five (6%); and others, 23 (27%).

### Bacterial Isolates

Microbiological cultures yielded 681 and 95 isolates of the family Enterobacterales recovered from FHs and ICPs, respectively. *E. coli* isolates of 425 (62.4%) were the most predominant, followed by *K. pneumoniae* isolates of 18 (2.6%) among FHs, whereas the predominant isolates among ICPs were *K. pneumoniae* accounting for 69 (72.6%), followed by *E. coli* of 16 (16.8%). A total of 36/681 and 87/95 were CRE isolates giving prevalence rates of 5.3 and 91.5% among FHs and ICPs, respectively. The breakdown of the CRE isolates among CRE colonized FHs and ICPs is shown in Table 2. *E. coli* isolates represent 15 (41.7%) and 15 (17.3%) (*p* = 0.004) among FHs and ICPs, respectively, in addition to *K. pneumoniae* isolates representing 8 (22.2%) and 65 (74.7%) (*p* = 0.001) among FHs and ICPs, respectively. Other isolates represent 13 (36%) and 7 (8%) (*p* = 0.001) among FHs and ICPs, respectively.

**TABLE 2:** Carbapenem-resistant Enterobacterales (CRE) isolates from food handlers and infected control patients.

**Bacteria**	**Number (%) of CRE isolates**		***p*-value**
	**FHs (*N* = 36)**	**ICPs (*N* = 87)**	
*Escherichia coli*	15 (41.7)	15 (17.3)	0.004*
*Klebsiella pneumoniae*	8 (22.2)	65 (74.7)	0.001*
*Enterobacter cloacae*	3 (8.3)	4 (4.7)	0.41
*Kluyvera* spp.	2 (5.5)	0 (0)	
*Citrobacter freundii*	1 (2.8)	1 (1.2)	0.51
*Citrobacter youngae*	1 (2.8)	0 (0)	
*Escherichia fergusonii*	1 (2.8)	0 (0)	
*Morganella morganii*	1 (2.8)	0 (0)	
*Pantoea* spp.	1 (2.8)	1 (1.2)	0.51
*Proteus vulgaris*	1 (2.8)	0 (0)	
*Providencia rettegri*	1 (2.8)	0 (0)	
*Serratia marcescens*	1 (2.8)	1 (1.2)	0.51

*FHs, food handlers; ICPs, infected control patients.*

*p-value is significant.

### Indirect Carbapenemase Test

**FIGURE 1:**
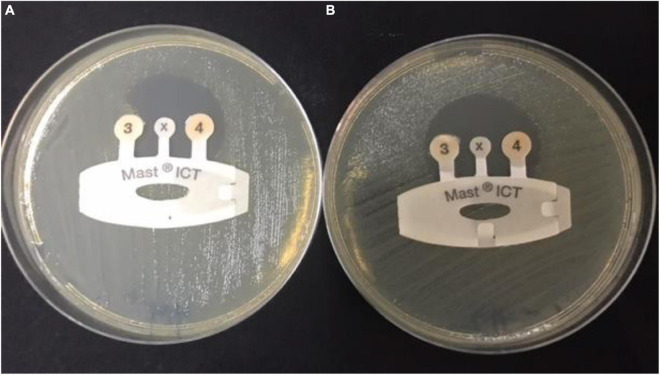
Detection of carbapenemases in carbapenem-resistant Enterobacteriaceae sample by indirect carbapenemase test (ICT). **(A)** Positive CRE sample: ICT positive, showing carbapenemase production and distortion of the zone around tip 3. **(B)** Negative sample: ICT negative, showing no carbapenemases production and formation of a regular, circular zone of inhibition around the indicator tip 3. CRE, carbapenem-resistant Enterobacterales.

All CRE isolates were tested with MAST^®^ Indirect Carbapenemase Test (ICT). Carbapenemase production was detected in 15 (41.6%) of the FHs (*n* = 36) and 44 (50.5%) of the control group (*n* = 87). Positive carbapenemase production was indicated when there is a distortion of the zone around the tip of the ICT, as shown in Figure 1.

### Carbapenemase Genes

Twenty-two (61.1%) out of 36 CRE isolates were recovered from FHs, and 65 (74.7%) out of 87 CRE isolates from ICPs harbored one, two, or three carbapenemase-mediating genes, as shown in Table 3. The detection of *bla* genes among FHs and ICPs was as follows: 54 CRE isolates harbored single genes, and of these, 15 (68.2%) and 39 (60%) isolates were from FHs and ICPs, respectively. In addition to the coexistence of two genes that were observed in 28 CRE isolates, of these, 5 (22.7%) and 23 (35.4%) were from FHs and ICPs, respectively. Five CRE isolates were in combination with three genes, and of these, 2 (9%) and 3 (4.6%) were from FHs and ICPs, respectively. There are no statistical differences between the FHs and ICPs groups.

**TABLE 3:** Prevalence of mediating carbapenem resistance genes in isolates from food handlers and infected control patients.

**Carbapenemase genes detection**	**Number (%) of CRE isolates**		
	**FHs (*N* = 36)**	**ICPs (*N* = 87)**	***p-*value**
**Total number of gene detected**	22 (61.1)	65 (74.7)	0.13
**Single gene**	15 (68.2)	39 (60)	0.49
*bla*_OXA_	3 (20)	21 (53.8)	0.05
*bla*_NDM_	0	10 (25.7)	
*bla*_KPC_	12 (80)	8 (20.5)	0.05
**Dual genes**	5 (22.7)	23 (35.4)	0.27
*bla*_OXA_/*bla*_KPC_	5 (100)	12 (52.2)	
*bla*_OXA_/*bla*_NDM_	0	8 (34.8)	
*bla*_NDM_/*bla*_KPC_	0	3 (13)	
**Triple genes**	2 (9)	3 (4.6)	
*bla*_OXA_/*bla*_NDM_/*bla*_KPC_			0.43

*FHs, food handlers; ICPs, infected control patients.*

In Table 4, the predominant CRE genes harbored by FHs 22/36 (61.1%) and ICPs 65/87 (74.7%) isolates were as follows: the occurrence of *bla*_OXA_ genes was observed in all isolates of FHs 10/22 (45.5%) whereas 44/65 (67.7%) isolates among ICPs (*p* = 0.06). The presence of *bla*_KPC_ genes was detected in 19/22 (86%) and 26/65 (40%) from FHs and ICPs (*p* = 0.0001), respectively. The presence of *bla*_NDM_ genes was identified in 2/22 (9.1%) and 24/65 (36.9%) isolates from FHs and ICPs (*p* = 0.01), respectively.

**TABLE 4:** Proportion of CRE isolates expressing carbapenemase gene variants among food handlers and infected control patients.

	**Number (%) of CRE isolates**		
**Carbapenemase genes**	**expressing carbapenemase genes**		
	**FHs**	**ICPs**	***p-*value**
**Overall isolates**	22 (61.1)	65 (74.7)	
*bla*_OXA_ (54)	10 (45.5)	44 (67.7)	0.06
*bla*_KPC_ (45)	19 (86.4)	26 (40)	0.0001*
*bla*_NDM_ (26)	2 (9.1)	24 (36.9)	0.01*
***Klebsiella pneumoniae*** (52)	7 (32)	45 (69)	0.004*
*bla*_OXA_			
*bla*_OXA–48_ (15)	5 (22.7)	10 (15.3)	0.006*
*bla*_OXA–181_ (18)	0	18 (27.6)	
*bla*_OXA–232_ (1)	0	1 (1.5)	
*bla*_KPC_			
*bla*_KPC_ (1)	1 (4.5)	0	
*bla*_KPC–18_ (5)	5 (22.7)	0	
*bla*_KPC–2_ (17)	3 (13.6)	14 (21.5)	0.4
*bla*_KPC–29_ (2)	0	2 (3)	
*bla*_KPC–20_ (0)	0	0	
*bla*_NDM_			
*bla*_NDM–1_ (18)	1 (4.5)	17 (26)	0.06
*bla*_NDM–5_ (0)	0	0	
*bla*_NDM–6_ (1)	0	1 (1.5)	
*bla*_NDM–7_ (1)	0	1 (1.5)	
***Escherichia coli*** (26)	11 (50)	15 (23)	0.01*
*bla*_OXA_			
*bla*_OXA–48_ (15)	4 (18)	11 (17)	0.8
*bla*_OXA–181_ (1)	0	1 (1.5)	
*bla*_OXA–232_ (0)	0	0	
*bla*_KPC_			
*bla*_KPC_ (4)	4 (18)	0	
*bla*_KPC–18_ (4)	0	4 (6)	
*bla*_KPC–2_ (1)	0	1 (1.5)	
*bla*_KPC–29_ (2)	2 (9)	0	
*bla*_KPC–20_ (2)	0	2 (3)	
*bla*_NDM_			
*bla*_NDM–1_ (3)	0	3 (4.6)	
*bla*_NDM–5_ (1)	0	1 (1.5)	
*bla*_NDM–6_ (1)	0	1 (1.5)	
*bla*_NDM–7_ (1)	1 (4.5)	0	
**Others** (9)*	4 (18)	5 (8)	0.16
*bla*_OXA_			
*bla*_OXA–48_ (3)	1 (4.5)	2 (3)	0.7
*bla*_OXA–181_ (1)	0	1 (1.5)	
*bla*_OXA–232_ (0)	0	0	
*bla*_KPC_			
*bla*_KPC_ (3)	3 (13.6)	0	
*bla*_KPC–18_ (0)	0	0	
*bla*_KPC–2_ (4)	1 (4.5)	3 (4.6)	0.9
*bla*_KPC–29_ (0)	0	0	
*bla*_KPC–20_ (0)	0	0	
*bla*_NDM_			
*bla*_NDM–1_ (0)	0	0	
*bla*_NDM–5_ (0)	0	0	
*bla*_NDM–6_ (0)	0	0	
*bla*_NDM–7_ (0)	0	0	

*CRE, carbapenem-resistant Enterobacterales; FHs, food handlers; ICPs, infected control patients.*

*Others = Enterobacter cloacae (5), Serratia marcescens (2), Pantoea (1), Citrobacter freundii (1), Kluyvera (1), and Escherichia fergusonii (1).

As demonstrated in Table 4, sequence analysis of *K. pneumoniae*, *E. coli*, and other isolates harboring *bla*_OXA_, *bla*_KPC_, and *bla*_NDM_ recovered from FHs and ICPs shows the following. Allelic variants of *bla*_OXA_ as *bla*_OXA–48_ genes were positive for 5/22 (22.7%) and 10/65 (15.3%) *K. pneumoniae* isolates from FHs and ICPs, respectively. Among ICPs alone, 18/65 (27.6%) and 1/65 (1.5%) *K. pneumoniae* isolates carried *bla*_OXA–181_ and *bla*_OXA–232_, respectively.

The allelic variants of *E. coli* isolates, 4/22 (18%) and 11/65 (17%), were harbored by *bla*_OXA–48_ genes among FHs and ICPs, respectively. One *E. coli* isolate out of 65 CRE isolates (1.5%) harbored *bla*_OXA–181_ gene among ICPs. The sequenced variants among other isolates showed that *bla*_OXA–48_ gene was detected in a single *Citrobacter freundii* isolate out of 22 CRE isolates (4.5%) from FHs and 2/65 (3%) *Enterobacter cloacae* and *Serratia marcescens* isolates from ICPs. In addition, *bla*_OXA–181_ was detected in an *E. cloacae* isolate out of 65 CRE isolates (1.5%) from ICPs.

Eleven (57.9%) of 19 randomly selected *bla*_KPC_-positive isolates from FHs were sequenced. *bla*_KPC–18_ genes were harbored by 5/22 (22.7%) *K. pneumoniae* isolates from FHs. In addition, 3/22 (13.6%) and 14/65 (21.5%) *K. pneumoniae* isolates carried *bla*_KPC–2_ gene from FHs and ICPs, respectively, while *bla*_KPC–29_ was detected in 2/65 (3%) *K. pneumoniae* isolates among ICPs only. The sequenced variants of *bla*_KPC_ among *E. coli* isolates showed the following: 4 (6%), 1 (1.5%), and 2 (3%) out of 65 CRE *E. coli* isolates harbored *bla*_KPC–18_, *bla*_KPC–2_, and *bla*_KPC–20_ among ICPs, respectively. However, *bla*_KPC–29_ was harbored by 2/22 (9%) *E. coli* isolates among FHs only. The sequenced variants of *bla*_KPC_ among other isolates showed the following: *bla*_KPC–2_ was harbored by 1/22 (4.5%) *E. cloacae* from FHs and 3/65 (4.6%) including *E. cloacae*, *S. marcescens*, and *Pantoea* isolates from ICPs.

The sequenced variants of the *bla*_NDM_ among *K. pneumoniae* isolates yielded *bla*_NDM–1_ gene carried by one *K. pneumoniae* isolate (4.5%) out of 22 CRE isolates from FHs and 17/65 (26%) from ICPs. Furthermore, one *K. pneumoniae* isolate (1.5%) carried *bla*_NDM–6_ and *bla*_NDM–7_ out of 65 CRE isolates from ICPs. Of the *E. coli* isolates that carried *bla*_NDM_ genes, 1/22 (4.5%) harbored *bla*_NDM–7_ among FHs only, while *bla*_NDM–1_, *bla*_NDM–5_, and *bla*_NDM–6_ were harbored by 3 (4.6%), 1 (1.5%), and 1 (1.5%) *E. coli* isolates out of 65 CRE isolates from ICPs alone, respectively.

### Clonal Relatedness of Isolates

**FIGURE 2:**
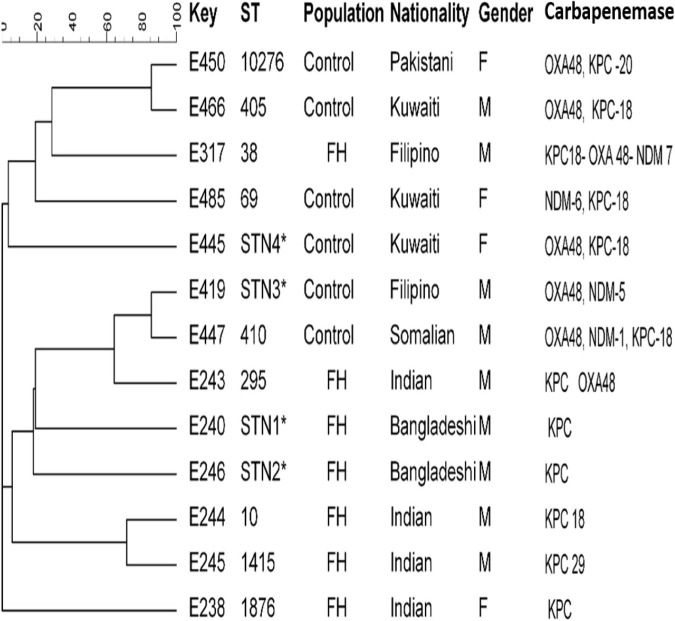
Dendrogram of *Escherichia coli* obtained from food handlers and infected control patients showing clonal relatedness demonstrated by cluster analysis based on their MLST. The characteristics of the major clones generated using minimum spanning trees by BioNumerics software v6.1 (Applied Maths, Kortrijk, Belgium) are also highlighted. Similarity in the isolates is presented in percentages using the scale bar in the upper left corner. In the Key column, E represents *E. coli*, STs = sequence type; population = food handler (FH) and infected control patients; nationality; gender F = female, male = male; and carbapenemases. MLST, multilocus sequence typing.

Clonal relationships of 27 CRE isolates that carried either *bla*_OXA_ or *bla*_KPC_ or *bla*_NDM_ genes from FHs and ICPs were genotyped by MLST. Figure 2 demonstrates the selected carbapenem-resistant *E. coli* isolates from FHs. Those isolates were found to belong to seven unique STs with the following types: ST38, ST295, ST10, ST1415, and ST1876. Those STs were considered genotypically distinct. In addition to two novel STs, STN1, and STN2 lineages were found for the first time in this study. The CCs assigned *E. coli* isolates of FHs into five CCs consisting of ST1876, which belongs to CC538; ST295, CC295; ST10, CC10; ST1415, CC1415; and ST38, CC38. Out of the six *E. coli* isolates analyzed among ICPs, four known ST types were yielded—ST10276, ST405, ST69, and ST410—besides two novel STs, STN3 and STN4. The four *E. coli* ST isolates were assigned into different CCs, as follows: ST410 to CC23; ST10276, CC405; ST405, CC405; and ST69, CC69. According to the dendrogram, two clones including ST type ST10276 and ST405 with similar CC (CC405) were considered to be closely related clones with the coexistence of *bla*_OXA_/*bla*_KPC_ carbapenemase genes. Another closely related two isolates belonged to the newly described ST types STN3 and ST410.

**FIGURE 3:**
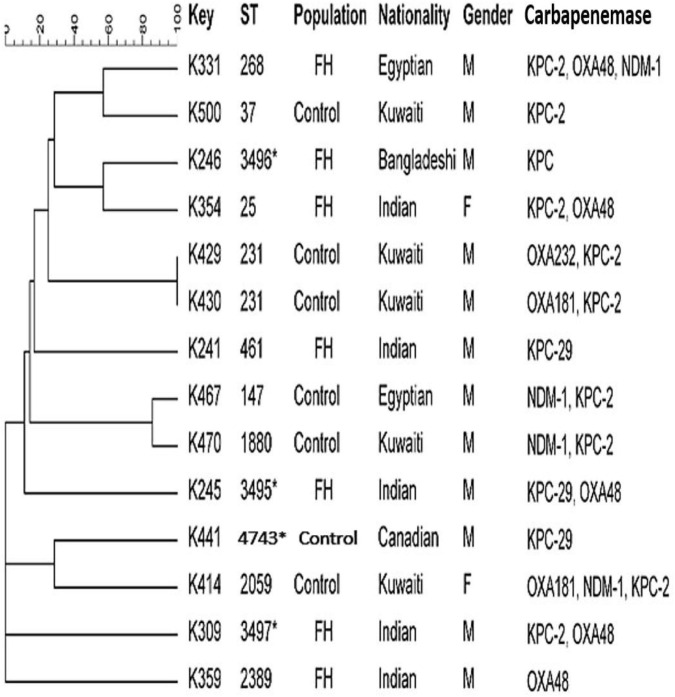
Dendrogram of *Klebsiella pneumoniae* obtained from food handlers and infected control patients showing clonal relatedness demonstrated by cluster analysis based on their MLST. The characteristics of the major clones generated using minimum spanning trees by BioNumerics software v6.1 (Applied Maths, Kortrijk, Belgium) are also highlighted. Similarity in the isolates is presented in percentages using the scale bar in the upper left corner. In the Key column, K represents *K. pneumoniae*, STs = sequence type; population = food handler (FH) and infected control patients; nationality; gender F = female, male = male; and carbapenemases. MLST, multilocus sequence typing.

Figure 3 shows that the MLST analysis of representative carbapenem-resistant *K. pneumoniae* isolates obtained from FHs revealed seven different STs. The four known ST types were as follows: ST461, ST268, ST25, and ST2389. In addition, three novel combinations of alleles and thus undescribed STs designated ST3495, ST3496, and ST3497 were assigned by Pasteur Institute MLST database. Those STs were considered genotypically distinct. The CCs assigned *K. pneumoniae* isolates into five CCs, as follows: ST461, CC461; ST3497, CC1155; ST268, CC268; ST25, CC65; and ST2389, CC2274. However, *K. pneumoniae* isolates obtained from ICPs revealed five different and known STs, which were as follows: ST37, ST2059, ST147, ST1880, and ST231. In addition to one novel ST identified for the first time in this study, submitted to the Pasteur Institute MLST scheme and given a new designation ST4743. The analysis of goeBURST assigned *K. pneumoniae* isolates into five CCs: ST37 belonged to CC37; ST2059, CC138; ST231, CC43; ST147, CC147; and ST1880, CC147. Two identical isolates that belonged to ST231 with CC43 were isolated from two Kuwaiti ICPs admitted to MAK Hospital; isolates K429 and K430 co-harbored dual genes *bla*_OXA–232_/*bla*_KPC–2_ and *bla*_OXA–181_/*bla*_KPC–2_, respectively. Moreover, there are two related isolates ST147 and ST1880, with similar CC (CC147) harboring in combination genes *bla*_NDM–1_ and *bla*_KPC–2_. Those isolates were isolated from Egyptian and Kuwaiti ICPs admitted to MAK Hospital.

## Discussion

In this study, we described the occurrence of the CRE isolates among FHs in our community. Unrecognized personnel working in commercial food services colonized with CRE and unsafe food handling could be potential sources of antimicrobial resistance dissemination. Molecular characteristics of the CRE isolates revealed that not all the isolates that were resistant to the carbapenems harbored the carbapenemase-encoding genes, demonstrated by the fact that only 61.1 and 74.7% of CRE were positive among the FHs and ICPs isolates, respectively. The most plausible explanation is that these negative CRE isolates probably expressed different resistance mechanisms other than carbapenemase-encoding genes not evaluated in this study. One of the most important findings of this study is the high preponderance of *bla*_KPC_, representing 86.4% of the genes found among FHs isolates with various variants like *bla*_KPC–18_, *bla*_KPC–2_, and *bla*_KPC–29_ for the first time in Kuwait and very uncommon in the neighboring countries. Previously, it has been shown that the majority of the genes described in clinical isolates from Kuwait and Gulf Cooperation Council (GCC) region were *bla*_OXA_ and *bla*_NDM_ and anecdotal reports of clinical isolates of KPC-producing CRE. Most of the patients from whom these carbapenemase genes were found have so far been patients transferred from hospitals abroad (Zowawi et al., 2013, 2014; Sonnevend et al., 2015). Encountering a large number of isolates harboring *bla*_*KPC*_ in the current study suggested that there might be wide dissemination of this gene in our country. This gene has been predominantly found in *K. pneumoniae* all over the world, but in our study, it was also found in other isolates such as *E. coli*, *E. cloacae*, *S. marcescens*, and *Pantoea*. This finding is similar to that obtained in previous studies that documented the presence of *bla*_KPC_ genes in many clinical isolates of *E. coli*, *Enterobacter* spp., *Salmonella enterica*, *Proteus mirabilis*, and *C. freundii* (Queenan and Bush, 2007).

Other interesting findings in our study included a high number of rectal isolates harboring *bla*_OXA–181_ gene among the ICP population as well as the FHs. This confirms an earlier report by Al Fadhli et al. (2020) heralding the emergence of this gene among the family Enterobacterales colonizing the gastrointestinal tract of patients in our hospitals, a phenomenon hitherto confined to the Indian subcontinent (Castanheira et al., 2011) from where it has apparently spread to other parts of the world (Rojas et al., 2017).

Interestingly, we detected five *K. pneumoniae* isolates co-producing *bla*_NDM–1_/*bla*_OXA–181_. Similarly, the presence of the combination of OXA-181 and NDM-5-producing CRE was reported in India in 2011 encountered in patients admitted to hospitals (Castanheira et al., 2011). Finding this combination in *K. pneumoniae*, like ours, has only been reported in two isolates carrying both *bla*_OXA–181_ and *bla*_NDM–1_ or *bla*_NDM–5_ isolated from epidemiologically unrelated patients in Singapore in 2013 (Balm et al., 2013). Furthermore, the occurrence of *bla*_OXA–181_ gene in association with other carbapenemase genes as *bla*_KPC–2_ in *K. pneumoniae* isolates was found in the present study. This is the first of such findings in Kuwait, which can be a serious concern. It should be noted that in our study, a novel milieu of OXA48-like carbapenemase, as OXA-232, was detected in *K. pneumoniae* isolates from Kuwaiti patients with ST231. This is in line with a previous report in South India that found *bla*_OXA–232_ variant in 35 (71%) *K. pneumoniae* isolates, and ST231 was the predominant ST in 22 isolates (45%) (Shankar et al., 2019).

According to previous reports from Kuwait, *bla*_NDM–1_ is by far the most prevalent gene mediating resistance to carbapenems in clinical isolates of CRE (Jamal et al., 2012, 2013, 2016). In our current study, a relatively high proportion of *K. pneumoniae* isolates in ICPs harbored this gene as well as a few *E. coli* isolates. However, this gene was found in only one isolate in FHs. It is conceivable that perhaps this gene is also confined to the hospital, where it is gradually being replaced by *bla*_OXA–181_. A few new variants of *bla*_NDM_, *bla*_NDM–5_, *bla*_NDM–6_, and *bla*_NDM–7_, were detected in a few of the *K. pneumoniae* and *E. coli* isolates, particularly among the ICP group. Thus, our finding is concordant with the reports of previous studies, which demonstrated that these variants are present in a low level in clinical isolates in the Arabian Peninsula (Pal et al., 2017). A spillover of this gene from the rectal site into clinical isolates may herald a dangerous resistance trend in the country.

MLST of *E. coli* CRE isolates showed polyclonality with a diverse set of known STs with their CCs circulating among FHs, and two novel STs linked with KPC-producing isolates from Bangladeshi FHs national were typed. These individuals had a history of travel during the last 3 months and lived in the same district in Kuwait. In this study, an isolate belonging to ST10 type detected in Indian FHs has been related to various diseases caused by Enteroaggregative *E. coli* (Chattaway et al., 2014) in the past. In addition, ST295, detected in this study, has also been previously described in a study in Nigeria by Adesina et al. (2019), associating this clone to MDR extra-intestinal pathogenic *E. coli*. In our study, isolates belonging to ST10276 type obtained from a 60-year-old Pakistani female patient admitted to MAK Hospital and ST405 isolated from a Kuwaiti male patient admitted to ISH were closely related to and shared the same CC405 with the coexistence of *bla*_OXA–48_ and *bla*_KPC–18_ carbapenemase genes. Other related isolates with the novel STN3 were isolated from a Filipino male patient and ST410 from a Somalian male patient admitted to MAK Hospital. The international clones ST38 and ST405 harboring triple carbapenemases *bla*_KPC–18_/*bla*_OXA–48_/*bla*_NDM–7_ genes were isolated from the ICP group in this study. This is in line with previous studies that reported *E. coli* isolates that belonged to ST38 and ST405 were encountered in patients in 2015 in Kuwait (Jamal et al., 2015) and Saudi Arabia (Alghoribi et al., 2015).

Carbapenem-resistant *K. pneumoniae* strains obtained from the ICP group were assigned to several STs with their CCs. It is noteworthy that there were two isolates from the ICP group in MAK Hospital with identical STs ST231 with CC43 carrying dual *bla*_OXA_/*bla*_KPC_ genes. There were also two related isolates with > 95% similarity, in the same hospital, that belonged to different ST types ST147 and ST1880, sharing the same CC147 that harbored dual *bla*_NDM_/*bla*_KPC_ genes. This suggested that clonal dissemination might have occurred. Thus, different STs appeared to carry diverse drug-resistant profiles. It is important to note that some STs found among the rectal isolates in our study were different from the previously described ST types, such as ST677, ST16, ST107, ST485, ST1593, ST1592, and ST1594, among clinical isolates circulating in Kuwait (Jamal et al., 2015). The novel ST type ST3496 that harbored *bla*_KPC_ gene was recognized for the first time in a Bangladeshi FH who had a history of travel to his home country during the previous 3 months. In addition, the novel ST4743 was recognized for the first time in a Canadian patient who was admitted to MAK Hospital. The diversity of clones could be related to an increasing number of expatriate employees from diverse geographical areas working in Kuwait coupled with their constant movement to and from other parts of the world. The diverse genetic background of these resistant genes is presumably related to importation from different geographical regions, mainly from workers from South Asia. Many of these workers work in food vending establishments, which may explain, in part, the possible introduction of CRE isolates harboring a variety of encoding genes into the population over time. Therefore, it is our speculation at this time that some clones are probably expanding among these healthy subjects who work in the same Governorate and a large population of clients who may carry such strains to others. Adding to that, an increased number of Kuwait nationals travel abroad seeking medical treatment and are often re-admitted to local hospitals upon their return. This also creates opportunities for importing different CRE clones from other countries, thereby expanding the CRE population in the country’s hospitals.

A limitation of this study is that we did not investigate all carbapenemase resistance genes and other mechanisms of resistance.

## Conclusion

In conclusion, the study contributes to our understanding of the molecular epidemiology of CRE in the community of Kuwait. Our study revealed high prevalence rates of CRE rectal colonization among FHs and ICPs. The commonest mediating genes were *bla*KPC among FH isolates and *bla*OXA-types among patients’ isolates. Therefore, the emergence of KPC-carrying Enterobacterales in the healthy human population in the food industry is an unusual finding representing the first of such findings in our country. These results raise significant public health concerns in Kuwait hospitals and the community and highlight the need for necessary vigilance to detect community-acquired CRE isolates. Emphasis on the importance of continuous surveillance of the CRE strains to detect the introduction of new strains into the community and healthcare systems to avoid a trend toward endemicity is highly recommended. Further studies involving whole-genome sequencing (WGS) analysis should help to unravel the other possible mechanisms of resistance.

## Data Availability Statement

The original contributions presented in the study are included in the article/supplementary material, further inquiries can be directed to the corresponding author/s.

## Ethics Statement

The studies involving human participants were reviewed and approved by Institutional approval was secured from the Joint Committee for the Protection of Human Subjects in Research, Health Sciences Center, Kuwait University before commencing the data collection. In addition, authorizing Medical Ethics Committee of the Food and Nutrition Administration, Ministry of Health, Kuwait (permit number. 299/2015) was granted. Specimens collection was conducted according to the Declaration of Helsinki and with particular institutional ethical and professional standards. Written informed consent was taken from all participants. The patients/participants provided their written informed consent to participate in this study.

## Author Contributions

OM, NA-S, and VR: conceptualization, methodology, software, formal analysis, investigation, resources, and visualization. NA-S and VR: validation, data curation, writing—review and editing, and supervision. OM: writing—original draft preparation. NA-S: project administration. NA-S and OM: funding acquisition. All authors have read and agreed to the published version of the manuscript.

## Conflict of Interest

The authors declare that the research was conducted in the absence of any commercial or financial relationships that could be construed as a potential conflict of interest.

## Publisher’s Note

All claims expressed in this article are solely those of the authors and do not necessarily represent those of their affiliated organizations, or those of the publisher, the editors and the reviewers. Any product that may be evaluated in this article, or claim that may be made by its manufacturer, is not guaranteed or endorsed by the publisher.

## Footnotes

1
www.ncbi.nlm.nih.gov

2
https://pubmlst.org/mlst/

3
https://bigsdb.pasteur.fr/klebsiella/

4
http://www.phyloviz.net/goeburst/

## References

[B1] Adesina T. Nwinyi O. De N. Akinnola O. Omonigbehin E. (2019). First detection of carbapenem-resistant *Escherichia fergusonii* strains harbouring beta-lactamase genes from clinical samples. *Pathogens (Basel, Switzerland)* 8 164–176. 10.3390/pathogens8040164 31557915 PMC6963453

[B2] Al Fadhli A. H. Jamal W. Y. Rotimi V. O. (2020). Prevalence of carbapenem-resistant *Enterobacteriaceae* and emergence of high rectal colonization rates of blaOXA-181-positive isolates in patients admitted to two major hospital intensive care units in Kuwait. *PLoS One* 15:e0241971. 10.1371/journal.pone.0241971 33201906 PMC7671514

[B3] Alghoribi M. F. Gibreel T. M. Farnham G. Al Johani S. M. Balkhy H. H. Upton M. (2015). Antibiotic-resistant ST38, ST131 and ST405 strains are the leading uropathogenic *Escherichia coli* clones in Riyadh, Saudi Arabia. *J. Antimicrob. Chemother.* 70 2757–2762. 10.1093/jac/dkv188 26183183

[B4] Balm M. N. La M. V. Krishnan P. Jureen R. Lin R. T. Teo J. W. (2013). Emergence of *Klebsiella pneumoniae* co-producing NDM-type and OXA-181 carbapenemases. *Clin. Microbiol. Infect.* 19 E421–E423. 23668475 10.1111/1469-0691.12247

[B5] Castanheira M. Deshpande L. M. Mathai D. Bell J. M. Jones R. N. Mendes R. E. (2011). Early dissemination of NDM-1-and OXA-181-producing *Enterobacteriaceae* in Indian hospitals: report from the SENTRY Antimicrobial Surveillance Program, 2006-2007. *Antimicrob. Agents Chemother.* 55 1274–1278. 10.1128/aac.01497-10 21189345 PMC3067112

[B6] Chattaway M. A. Jenkins C. Rajendram D. Cravioto A. Talukder K. A. Dallman T. (2014). Enteroaggregative *Escherichia coli* have evolved independently as distinct complexes within the *E. coli* population with varying ability to cause disease. *PLoS One* 9:e112967. 10.1371/journal.pone.0112967 25415318 PMC4240581

[B7] Cienfuegos-Gallet A. V. Ocampo de Los Ríos A. M. Sierra Viana P. Ramirez Brinez F. Restrepo Castro C. Roncancio Villamil G. (2019). Risk factors and survival of patients infected with carbapenem-resistant *Klebsiella pneumoniae* in a KPC endemic setting: a case-control and cohort study. *BMC Infect. Dis.* 19:830. 10.1186/s12879-019-4461-x 31590648 PMC6781339

[B8] CLSI [Clinical and Laboratory Standard Institute] (2018). *Performance Standards for Antimicrobial Susceptibility Testing, Twenty-Eighth Informational Supplement CLSI publication 2018; M100–S28.* Wayne, PA: CLSI.

[B9] Diancourt L. Passet V. Verhoef J. Grimont P. A. Brisse S. (2005). Multilocus sequence typing of *Klebsiella pneumoniae* nosocomial isolates. *J. Clin. Microbiol.* 43 4178–4182. 10.1128/jcm.43.8.4178-4182.2005 16081970 PMC1233940

[B10] Ellem J. Partridge S. R. Iredell J. R. (2011). Efficient direct extended-spectrum β-lactamase detection by multiplex real-time PCR: accurate assignment of phenotype by use of a limited set of genetic markers. *J. Clin. Microbiol.* 49 3074–3077. 10.1128/jcm.02647-10 21613435 PMC3147779

[B11] Jamal W. Y. Albert M. J. Rotimi V. O. (2016). High prevalence of New Delhi metallo-β-lactamase-1 (NDM-1) producers among carbapenem-resistant *Enterobacteriaceae* in Kuwait. *PLoS One* 11:e0152638. 10.1371/journal.pone.0152638 27031521 PMC4816385

[B12] Jamal W. Y. Albert M. J. Khodakhast F. Poirel L. Rotimi V. O. (2015). Emergence of new sequence type OXA-48 carbapenemase-producing *Enterobacteriaceae* in Kuwait. *Microb. Drug Resist.* 21 329–334. 10.1089/mdr.2014.0123 25551428

[B13] Jamal W. Rotimi V. O. Albert M. J. Khodakhast F. Nordmann P. Poirel L. (2013). High prevalence of VIM-4 and NDM-1 metallo-β-lactamase among carbapenem-resistant *Enterobacteriaceae*. *J. Med. Microbiol.* 62 1239–1244. 10.1099/jmm.0.059915-0 23639985

[B14] Jamal W. Rotimi V. Albert M. J. Khodakhast F. Udo E. Poirel L. (2012). Emergence of nosocomial New Delhi metallo-β-lactamase-1 (NDM-1)-producing *Klebsiella pneumoniae* in patients admitted to a tertiary care hospital in Kuwait. *Int. J. Antimicrob. Agents* 39 177–185. 22113192 10.1016/j.ijantimicag.2011.10.002

[B15] Lee K. Lee W. G. Uh Y. Ha G. Y. Cho J. Chong Y. (2003). VIM- and IMP-type metallo-beta-lactamase-producing *Pseudomonas* spp. and *Acinetobacter* spp. in Korean hospitals. *Emerg. Infect. Dis.* 9 868–871. 12890331 10.3201/eid0907.030012 PMC3023439

[B16] Luo Y. Cui S. Li J. Yang J. Lin L. Hu C. (2011). Characterization of *Escherichia coli* isolates from healthy food handlers in hospital. *Microb. Drug Resist.* 17 443–448. 21612511 10.1089/mdr.2011.0032

[B17] Moghnia O. H. Rotimi V. O. Al-Sweih N. A. (2021a). Evaluating food safety compliance and hygiene practices of food handlers working in community and healthcare settings in Kuwait. *Int. J. Environ. Res.d Public Health* 18:1586. 10.3390/ijerph18041586 33567499 PMC7915981

[B18] Moghnia O. H. Rotimi V. O. Al-Sweih N. A. (2021b). Monitoring antibiotic resistance profiles of faecal isolates of *Enterobacteriaceae* and the prevalence of carbapenem-resistant isolates among food handlers in Kuwait. *J. Glob. Antimicrob. Resist.* 25 370–376. 10.1016/j.jgar.2021.04.009 33991748

[B19] Munoz-Price L. S. Poirel L. Bonomo R. A. Schwaber M. J. Daikos G. L. Cormican M. (2013). Clinical epidemiology of the global expansion of *Klebsiella pneumoniae* carbapenemases. *Lancet Infect. Dis.* 13 785–796. 10.1016/s1473-3099(13)70190-7 23969216 PMC4673667

[B20] Nordmann P. Poirel L. (2014). The difficult-to-control spread of carbapenemase producers among *Enterobacteriaceae* worldwide. *Clin. Microbiol. Infect.* 20 821–830. 10.1111/1469-0691.12719 24930781

[B21] Pal T. Ghazawi A. Darwish D. Villa L. Carattoli A. Hashmey R. (2017). Characterization of NDM-7 carbapenemase-producing *Escherichia coli* isolates in the Arabian Peninsula. *Microb. Drug Resist.* 23 871–878. 10.1089/mdr.2016.0216 28156193

[B22] Poirel L. Walsh T. R. Cuvillier V. Nordmann P. (2011). Multiplex PCR for detection of acquired carbapenemase genes. *Diagn. Microbiol. Infect. Dis.* 70 119–123. 10.1016/j.diagmicrobio.2010.12.002 21398074

[B23] Queenan A. M. Bush K. (2007). Carbapenemases: the versatile beta-lactamases. *Clin. Microbiol. Rev.* 20 440–458. 10.1128/cmr.00001-07 17630334 PMC1932750

[B24] Rojas L. J. Hujer A. M. Rudin S. D. Wright M. S. Domitrovic T. N. Marshall S. H. (2017). NDM-5 and OXA-181 beta-lactamases, a significant threat continues to spread in the americas. *Antimicrob. Agents. Chemother.* 61:e00454–17. 28461314 10.1128/AAC.00454-17 PMC5487671

[B25] Shankar C. Mathur P. Venkatesan M. Pragasam A. K. Anandan S. Khurana S. (2019). Rapidly disseminating blaOXA-232 carrying *Klebsiella pneumoniae* belonging to ST231 in India: multiple and varied mobile genetic elements. *BMC Microbiol.* 19:137. 10.1186/s12866-019-1513-8 31234800 PMC6591861

[B26] Sonnevend A. Ghazawi A. A. Hashmey R. Jamal W. Rotimi V. O. Shibl A. M. (2015). Characterization of carbapenem-resistant *Enterobacteriaceae* with high rate of autochthonous transmission in the Arabian Peninsula. *PLoS One* 10:e0131372. 10.1371/journal.pone.0131372 26110660 PMC4482506

[B27] Stoesser N. Sheppard A. E. Peirano G. Anson L. W. Pankhurst L. Sebra R. (2017). Genomic epidemiology of global *Klebsiella pneumoniae* carbapenemase (KPC)-producing *Escherichia coli*. *Sci. Rep.* 7 5917–5927. 28725045 10.1038/s41598-017-06256-2 PMC5517641

[B28] Temkin E. Adler A. Lerner A. Carmeli Y. (2014). Carbapenem-resistant *Enterobacteriaceae*: biology, epidemiology, and management. *Ann. N. Y. Acad. Sci.* 1323 22–42. 10.1111/nyas.12537 25195939

[B29] Toleman M. A. Biedenbach D. Bennett D. Jones R. N. Walsh T. R. (2003). Genetic characterization of a novel metallo-β-lactamase gene, bla IMP-13, harboured by a novel Tn 5051-type transposon disseminating carbapenemase genes in Europe: report from the SENTRY worldwide antimicrobial surveillance programme. *J. Antimicrob. Chemother.* 52 583–590. 10.1093/jac/dkg410 12951335

[B30] Wirth T. Falush D. Lan R. Colles F. Mensa P. Wieler L. H. (2006). Sex and virulence in *Escherichia coli*: an evolutionary perspective. *Mol. Microbiol.* 60 1136–1151. 10.1111/j.1365-2958.2006.05172.x 16689791 PMC1557465

[B31] Zowawi H. M. Balkhy H. H. Walsh T. R. Paterson D. L. (2013). β-Lactamase production in key gram-negative pathogen isolates from the Arabian Peninsula. *Clin. Microbiol. Rev.* 26 361–380. 10.1128/cmr.00096-12 23824364 PMC3719487

[B32] Zowawi H. M. Sartor A. L. Balkhy H. H. Walsh T. R. Al Johani S. M. Al Jindan R. Y. (2014). Molecular characterization of carbapenemase-producing *Escherichia coli* and *Klebsiella pneumoniae* in the countries of the Gulf cooperation council: dominance of OXA-48 and NDM producers. *Antimicrob. Agents Chemother.* 58 3085–3090. 10.1128/aac.02050-13 24637692 PMC4068443

